# Stability Analysis in Milling Based on the Localized Differential Quadrature Method

**DOI:** 10.3390/mi15010054

**Published:** 2023-12-26

**Authors:** Yonggang Mei, Bingbing He, Shangwen He, Xin Ren

**Affiliations:** 1 School of Construction Machinery, Chang’an University, Xi’an 710064, China; meiyonggang@chd.edu.cn; 2 College of Mechanical & Electrical Engineering, Shaanxi University of Science & Technology, Xi’an 710021, China; rxin0719@sohu.com; 3 School of Aeronautics, Northwestern Polytechnical University, Xi’an 710072, China; 4 School of Mechanics and Safety Engineering, Zhengzhou University, Zhengzhou 450001, China

**Keywords:** chatter, stability analysis, milling, localized differential quadrature method, stability lobe diagram

## Abstract

Chatter stability analysis is an effective way to optimize the cutting parameters and achieve chatter-free machining. This paper proposes a milling chatter stability analysis method based on the localized differential quadrature method (LDQM), which has the advantages of simple principle, easy application, and high computational efficiency. The milling process, considering the regeneration effect, is modeled using linear periodic delay differential equations (DDE), then the state transition matrix during the adjacent cutting period is constructed based on the LDQM, and finally, the stability of the milling process is obtained based on the Floquet theory. The accuracy and computation efficiency of the proposed method are verified through two benchmark milling models. The simulation results demonstrate that the proposed method in this paper can accurately and quickly obtain the chatter stability lobe diagram (SLD) of the milling process, which will provide guidance for optimizing the process parameters.

## 1. Introduction

Chatter is a common problem in the cutting processes, which seriously reduces the machining accuracy and material removal rate. Therefore, achieving chatter-free cutting has always been an important research topic in the field of machining. Over the years, researchers have conducted a large number of studies on the generation mechanism of chatter [1,2,3,4,5], monitoring and identification of chatter [6,7,8,9], and suppression and elimination of chatter [10,11,12,13]. In these studies, various strategies for achieving chatter-free cutting have been proposed, among which optimizing process parameters to achieve chatter-free cutting is undoubtedly a low-cost and easy-to-implement approach.

The SLD reveals the chatter stability limits under different process parameters, which can be utilized to optimize the process parameters effectively. Researchers have proposed various methods to construct the cutting chatter SLD. Based on in-depth research on the stability of the cutting processes, Altintas and Budak proposed the single-frequency solution [14] and multi-frequency solution [15,16] methods. These frequency domain methods have subsequently been widely used for the cutting stability prediction. Bayly et al. [17] divided the vibration state of the tool during the milling processes into two types: the free vibration and the forced vibration, and derived the temporal finite element analysis (TFEA) method, which can be used to predict the stability for arbitrary times in the cut. Butcher et al. introduced the Chebyshev polynomial method [18] and the Chebyshev collocation method [19] for milling stability analysis. Insperger et al. [20,21] put forward the semi-discretization method (SDM) to study the delayed system and adopted the zeroth-order SDM for milling stability analysis. Afterwards, Insperger et al. [22] proposed the first-order SDM (1st-SDM) and higher-order SDM, and used these methods to construct the stability chart of the delayed Mathieu equation. From the viewpoint of modern numerical analysis, the SDM and TFEA were based on the differential equation theory and the variational method respectively [23]. Although the SDM and TFEA can achieve accurate results, their computational efficiency is not high. When using these methods for the cutting stability analysis, a significant amount of computational time is often required. On account of the direct integration scheme, Ding et al. [24] developed a full-discretization method (FDM) to predict the stability of milling processes. The simulation calculation results indicated that the FDM has high computational efficiency without loss of numerical precision. Later, Ding et al. [25] proposed the numerical integration method for chatter stability analysis. In the numerical integration method, Ding et al. used the trapezoidal rule, Simpson’s rule, and Gaussian formula to approximate the integral term in the solution vector. Finally, the Floquet transition matrix of the milling processes was constructed. On the basis of Runge–Kutta methods, Niu et al. [26] proposed the classical fourth-order Runge–Kutta method and the generalized Runge–Kutta method to predict the stability of the milling processes, taking the regenerative effect into account. Zhang et al. [27] explored a Simpson-based method to predict the milling stability. The milling dynamic process is described as a linear time-periodic system with a single discrete time delay. Then, the Simpson method is utilized in each time interval to estimate the state items. Finally, the state transition matrix over one tooth passing period is constructed. Based on the linear multistep method, Mei et al. [28] developed a cutting chatter stability prediction method and investigated the stability of the milling processes with single delay. Later, Mei et al. [29] proposed an adaptive variable-step numerical integration method to investigate the stability of the milling process with multiple delays. The adaptive variable-step numerical integration method takes into account the effect of the helix angle and improves the discretization accuracy of the cutting period, and thus improves the calculation accuracy of the milling stability limit. Ma et al. [30] presented an updated FDM for milling stability prediction based on cubic spline interpolation. Zheng et al. [31] proposed a numerical method based on the composited Newton–Cotes formula to predict milling stability.

In summary, it can be observed that the main work of chatter stability analysis or prediction is to develop various algorithms to solve the cutting dynamics equation and construct the Floquet transition matrix. Therefore, constructing efficient numerical algorithms is crucial for chatter stability analysis. The differential quadrature method (DQM) is a numerical discretization technique that was first employed to solve partial differential equations by Bellman R. et al. [32,33]. Bert C W. et al. [34] applied the DQM to structural vibration analysis and confirmed that this method has the advantages of simple formula and low computational complexity. Ding et al. studied the stability of milling systems with single delay [35] and multiple delays [36] using the DQM. Despite the advantages mentioned above, the classical DQM also has its shortcomings. For example, the classical DQM is sensitive to the distribution of discrete nodes, and it is prone to generating ill-conditioned coefficient matrix when the number of discrete nodes is large. In order to improve these shortcomings, Zong Z. et al. [37] introduced the LDQM and successfully applied it to the problem of two-dimensional wave equations. Tsai C H. et al. [38] employed the LDQM to solve two-dimensional stream function formulation of incompressible Navier–Stokes equations. Inspired by these works, this paper proposes a milling chatter stability analysis method based on the LDQM.

This paper is organized as follows: In Section 2, the basic dynamics model of the milling process considering the regeneration effect is outlined. In Section 3, the basic theory of DQM is summarized and the algorithm for stability analysis based on the LDQM is formulated. The effectiveness of the proposed method is verified in Section 4, and the verification results are discussed. Finally, conclusions are derived in Section 5.

## 2. Dynamics Model of the Milling Process

**Figure 1:**
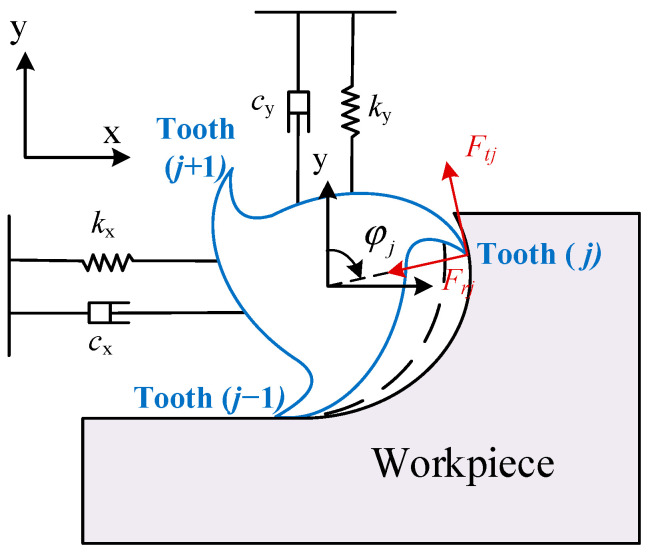
Schematic of the two-DOF milling system.

The milling dynamics model is the basis for studying the stability of the milling process. In this Section, a two-degree of freedom (DOF) milling system (as shown in Figure 1) is used to derive the dynamics model of the milling process. In Figure 1, $F_{tj}$ and $F_{rj}$ are the tangential and normal cutting force components acting on the cutting tooth ( j ), respectively. $\varphi_{j}$ is the angular position of the tooth ( j ) and can be calculated by the following formula:

(1) $$\varphi_{j}\left(t\right)=\varphi_{1}\left(0\right)-\left(j-1\right)\cdot \frac{2\pi }{N}+\frac{2\pi \cdot \Omega }{60}\cdot t$$

where Ω is the spindle speed and N is the number of cutting teeth.

Based on the linear cutting force model, the milling process considering the regeneration effect is modeled using a two-DOF DDE, which can be written as

(2) $$M\ddot{y}\left(t\right)+C\dot{y}\left(t\right)+Ky\left(t\right)=w\cdot H_{d}\left(t\right)\cdot \left[y\left(t\right)-y\left(t-T\right)\right]$$

where M , C , and K are the mass, damping, and stiffness matrices of the milling system, respectively, the values of which can be obtained through dynamic experiments; $y\left(t\right)={\left[\begin{array}{cc} x\left(t\right) & y\left(t\right) \end{array}\right]}^{T}$ and $y\left(t-T\right)={\left[\begin{array}{cc} x\left(t-T\right) & y\left(t-T\right) \end{array}\right]}^{T}$ are the vibration displacements at the current moment and the previous tooth passing period separately; w is the axial depth of cutting; T=60/(Ω⋅N) is the time delay that equals to the tooth passing period. $H_{d}\left(t\right)$ is the time-varying cutting force coefficients matrix with the period of T . Matrix $H_{d}\left(t\right)$ can be formulated as

(3) $$H_{d}\left(t\right)=\left[\begin{array}{cc} h_{xx}\left(t\right) & h_{xy}\left(t\right)\\ h_{yx}\left(t\right) & h_{yy}\left(t\right) \end{array}\right]$$

where

(4) $$\{\begin{array}{c} h_{xx}\left(t\right)=\sum_{j=1}^{N}g_{j}\left(t\right)\cdot \left(-\cos\varphi_{j}\left(t\right)\sin\varphi_{j}\left(t\right)K_{\mathrm{tc}}-\sin^{2}\varphi_{j}\left(t\right)K_{\mathrm{rc}}\right)\\ h_{xy}\left(t\right)=\sum_{j=1}^{N}g_{j}\left(t\right)\cdot \left(-\cos^{2}\varphi_{j}\left(t\right)K_{\mathrm{tc}}-\sin\varphi_{j}\left(t\right)\cos\varphi_{j}\left(t\right)K_{\mathrm{rc}}\right)\\ h_{yx}\left(t\right)=\sum_{j=1}^{N}g_{j}\left(t\right)\cdot \left(+\sin^{2}\varphi_{j}\left(t\right)K_{\mathrm{tc}}-\cos\varphi_{j}\left(t\right)\sin\varphi_{j}\left(t\right)K_{\mathrm{rc}}\right)\\ h_{yy}\left(t\right)=\sum_{j=1}^{N}g_{j}\left(t\right)\cdot \left(+\sin\varphi_{j}\left(t\right)\cos\varphi_{j}\left(t\right)K_{\mathrm{tc}}-\cos^{2}\varphi_{j}\left(t\right)K_{\mathrm{rc}}\right) \end{array}$$

In Equation (4), $K_{\mathrm{tc}}$ and $K_{\mathrm{rc}}$ are the linear cutting force coefficients in the tangential and radial directions, respectively, and $g_{j}\left(t\right)$ is a window function used to determine whether the cutter teeth (j) participate in cutting. Equation (2) can be converted to the state-space form as

(5) $$\dot{x}\left(t\right)=Ax\left(t\right)+B\left(t\right)\left[x\left(t\right)-x\left(t-T\right)\right]$$

where A is a constant matrix, while B is a periodic matrix satisfying B(t)=B(t+T) , and x is the state vector. A , B , and x can be represented as follows:

(6) $$A=\left[\begin{array}{cc} 0 & I\\ -M^{-1}K & -M^{-1}C \end{array}\right]$$

(7) $$B\left(t\right)=\left[\begin{array}{cc} 0 & 0\\ w\cdot M^{-1}H_{d}\left(t\right) & 0 \end{array}\right]$$

(8) $$x\left(t\right)=\left[\begin{array}{c} y\left(t\right)\\ \dot{y}\left(t\right) \end{array}\right]$$

where 0 and I are zero and identity matrix with the same dimension as M , the superscript ‘−1’ indicates the operation of matrix inversion.

From a mathematical point of view, Equation (5) describes a linear periodic system. The stability of the linear periodic system can be determined through the Floquet theory. According to the Floquet theory, the linear periodic system is stable if and only if the spectral radius of its Floquet transition matrix is less than one.

## 3. Algorithm Derivation

### 3.1. Classical Differential Quadrature Method

DQM is a numerical discretization technique used to solve the numerical solution of differential equations. Essentially, the classical DQM replaces the derivative of the function at each node with the weighted sum of the function values at all nodes in the calculation area. Then, the differential equations have been converted into algebraic equations which can be easily solved.

**Figure 2:**
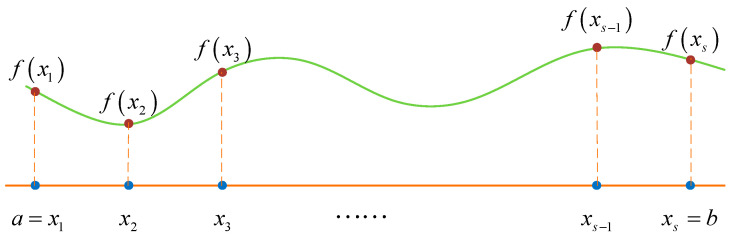
Function interpolation on interval [*a*, *b*].

Here, we take one-dimensional space as an example to briefly introduce the DQM. As shown in Figure 2, f(x) is a continuous differentiable function defined on the interval [a,b] , which is divided by s nonoverlapping nodes. According to the interpolation theory, we can get

(9) $$f\left(x\right)=\sum_{j=1}^{s}p_{j}\left(x\right)f\left(x_{j}\right)$$

where $p_{j}\left(x\right)$ is called the interpolation basis function.

Taking the k-th derivative of x on both sides of Equation (9) and substituting $x=x_{i}$ can obtain the k-th derivative of the function f(x) at the node $x_{i}$ .

(10) $${\frac{d^{k}\left(f\left(x\right)\right)}{dx^{k}}|}_{x=x_{i}}=\sum_{j=1}^{s}\left[{\frac{d^{k}\left(p_{j}\left(x\right)\right)}{dx^{k}}|}_{x=x_{i}}\cdot f\left(x_{j}\right)\right]$$

For the convenience of description, let $f_{i}^{\left(k\right)}$ denote ${\frac{d^{k}\left(f\left(x\right)\right)}{dx^{k}}|}_{x=x_{i}}$ , $W_{ij}^{\left(k\right)}$ denote ${\frac{d^{k}\left(p_{j}\left(x\right)\right)}{dx^{k}}|}_{x=x_{i}}$ , and $f_{j}$ denote $f\left(x_{j}\right)$ in the remainder of this work. Hence, Equation (10) can be expressed as

(11) $$f_{i}^{\left(k\right)}=\sum_{j=1}^{s}W_{ij}^{\left(k\right)}\cdot f_{j}$$

where $W_{ij}^{\left(k\right)}$ is the weighted coefficient. Applying the rule given in Equation (11) to all nodes on the interval [a,b] , we can obtain the following expression.

(12) $$F^{\left(k\right)}=W^{\left(k\right)}\cdot F$$

where

(13) $$F^{\left(k\right)}={\left[\begin{array}{cccc} f_{1}^{\left(k\right)} & f_{2}^{\left(k\right)} & \cdots & f_{s}^{\left(k\right)} \end{array}\right]}^{T}$$

(14) $$W^{\left(k\right)}=\left[\begin{array}{cccc} W_{11}^{\left(k\right)} & W_{12}^{\left(k\right)} & \cdots & W_{1s}^{\left(k\right)}\\ W_{21}^{\left(k\right)} & W_{22}^{\left(k\right)} & \cdots & W_{2s}^{\left(k\right)}\\ \vdots & \vdots & \ddots & \vdots \\ W_{s1}^{\left(k\right)} & W_{s2}^{\left(k\right)} & \cdots & W_{ss}^{\left(k\right)} \end{array}\right]$$

(15) $$F={\left[\begin{array}{cccc} f_{1} & f_{2} & \cdots & f_{s} \end{array}\right]}^{T}$$

Then, the rules given in Equation (12) can be used to conveniently solve the differential equations numerically.

It can be inferred from the derivation process that the core work of DQM is to obtain the weighted coefficient matrix W . Obviously, the calculation of weight coefficients depends on the selection of interpolation basis functions. The commonly used basis functions include Lagrange basis functions, exponential basis functions, harmonic interpolation basis functions, etc. In this work, Lagrange basis functions are adopted for algorithm derivation. Hence, $p_{j}\left(x\right)$ in Equation (9) can be represented as

(16) $$p_{j}\left(x\right)=\prod_{\begin{array}{l} r=1\\ r\neq j \end{array}}^{s}\frac{\left(x-x_{r}\right)}{\left(x_{j}-x_{r}\right)}$$

Then, the elements in the weighted coefficient matrix W can be expressed as

(17) $${Wi}_{j}=\{\begin{array}{l} \frac{\prod_{\begin{array}{l} r=1\\ r\neq i,j \end{array}}^{s}\left(x_{i}-x_{r}\right)}{\prod_{\begin{array}{l} r=1\\ r\neq j \end{array}}^{s}\left(x_{j}-x_{r}\right)},j\neq i\\ \sum_{\begin{array}{l} r=1\\ r\neq i \end{array}}^{s}\frac{1}{\left(x_{i}-x_{r}\right)},j=i \end{array}$$

### 3.2. Stability Analysis of the Milling Process Based on DQM

In order to apply the Floquet theory for stability analysis, the Floquet transition matrix over one tooth passing period T needs to be determined. Similar to the TFEA method, we denoted $t_{0}$ as the time the cutter leaves the workpiece, $t_{f}$ as the duration of the free vibration, and $t_{c}$ as the duration of the forced vibration. During the free vibration duration $\left[t_{0},t_{0}+t_{f}\right]$ , the cutter leaves the workpiece and the periodic matrix B(t) in Equation (5) equal to zero. Hence, the state vector at the end of the free vibration has an analytical solution, which can be expressed as

(18) $$x\left(t_{0}+t_{f}\right)=e^{A\cdot t_{f}}\cdot x\left(t_{0}\right)$$

In the forced vibration duration, the state vector has no analytical solution and needs to be solved numerically. For the convenience of the numerical solution, we discretize the forced vibration duration into m parts with m+1 discrete nodes. According to the DQM [39], the derivative value $\dot{x}\left(t\right)$ on the time node $t_{i}$ can be expressed by the weighted sum of the function values x(t) at all discrete time nodes $t_{j}$ , j=1,2,⋯,m+1 , as shown in the following equation:

(19) $$\dot{x}\left(t_{i}\right)=\sum_{j=1}^{m+1}W_{i,j}\cdot x\left(t_{j}\right)$$

where $W_{i,j}=W_{i,j}⊗I$ , and ⊗ denote the Kronecker product. Thus, we can obtain the following representation during one principal period:

(20) $$\left[\begin{array}{c} \dot{x}\left(t_{1}\right)\\ \dot{x}\left(t_{2}\right)\\ \vdots \\ \dot{x}\left(t_{m+1}\right) \end{array}\right]=W\cdot \left[\begin{array}{c} x\left(t_{1}\right)\\ x\left(t_{2}\right)\\ \vdots \\ x\left(t_{m+1}\right) \end{array}\right]$$

where W is the weighted coefficient matrix, which can be expressed as

(21) $$W=\left[\begin{array}{cccc} W_{1,1} & W_{1,2} & \cdots & W_{1,m+1}\\ W_{2,1} & W_{2,2} & \cdots & W_{2,m+1}\\ \vdots & \vdots & \ddots & \vdots \\ W_{m+1,1} & W_{m+1,2} & \cdots & W_{m+1,m+1} \end{array}\right]$$

Substituting Equation (19) into Equation (5) and let $x_{i}$ denote $x\left(t_{i}\right)$ , $x_{i-T}$ denote $x\left(t_{i}-T\right)$ , $B_{i}$ denote $B\left(t_{i}\right)$ , we get

(22) $$\sum_{j=1}^{m+1}W_{i,j}\cdot x_{j}=Ax_{i}+B_{i}\left(x_{i}-x_{i-T}\right)$$

Applying Equation (22) at all the discrete time nodes, the following discrete dynamical map can be obtained.

(23) $$P_{1}\cdot {\left[\begin{array}{ccccc} x_{1} & x_{2} & x_{3} & \cdots & x_{m+1} \end{array}\right]}^{T}=Q_{1}\cdot {\left[\begin{array}{ccccc} x_{1-T} & x_{2-T} & x_{3-T} & \cdots & x_{m+1-T} \end{array}\right]}^{T}$$

Then, the Floquet transition matrix $\Phi_{1}$ based on the DQM over one tooth passing period T can be expressed as

(24) $$\Phi_{1}=P_{1}^{-1}\cdot Q_{1}$$

where

(25) $$P_{1}=\left[\begin{array}{cccc} A+B_{1}-W_{1,1} & -W_{1,2} & \cdots & -W_{1,m+1}\\ -W_{2,1} & A+B_{2}-W_{2,2} & \cdots & -W_{2,m+1}\\ \vdots & \vdots & \ddots & \vdots \\ -W_{m+1,1} & -W_{m+1,2} & \cdots & A+B_{m+1}-W_{m+1,m+1} \end{array}\right]$$

(26) $$Q_{1}=\left[\begin{array}{cccc} B_{1} & & & \\ & B_{2} & & \\ & & \ddots & \\ & & & B_{m+1} \end{array}\right]$$

According to the implicit relationship in Equation (18), matrices $P_{1}$ and $Q_{1}$ can be further rewritten as

(27) $$P_{1}=\left[\begin{array}{cccc} I & 0 & \cdots & 0\\ -W_{2,1} & A+B_{2}-W_{2,2} & \cdots & -W_{2,m+1}\\ \vdots & \vdots & \ddots & \vdots \\ -W_{m+1,1} & -W_{m+1,2} & \cdots & A+B_{m+1}-W_{m+1,m+1} \end{array}\right]$$

(28) $$Q_{1}=\left[\begin{array}{cccc} 0 & 0 & \cdots & e^{A\cdot t_{f}}\\ & B\left(t_{2}\right) & & \\ & & \ddots & \\ & & & B\left(t_{m+1}\right) \end{array}\right]$$

The stability of the milling process can be determined using the spectral radius of the Floquet transition matrix $\Phi_{1}$ . It can be seen from Equation (21) that the coefficient matrix of the classical DQM is a full matrix. Once the selection of discrete nodes is unreasonable or the number is large, it is prone to generate an ill-conditioned coefficient matrix, which can bring catastrophic consequences to the subsequent numerical calculations. To improve this deficiency, we propose a novel chatter stability analysis method based on the LDQM, and the specific derivation process of the algorithm is provided in the next Section.

### 3.3. A Novel Chatter Stability Analysis Method Based on the LDQM

In the LDQM, the derivative of the function at each node is represented only by the weighted sum of function values of some nearby nodes. In the calculation process of the weighted coefficient matrix, low-order polynomial interpolation is used, which will generate a sparse banded coefficient matrix. Compared with dense matrices, sparse matrices have better storage and operational characteristics which provides the possibility to solve large-scale engineering problems.

According to the idea of LDQM, we select l ( l≪m+1 ) nodes near the i-th node to calculate the weighted coefficients, rather than select all the m+1 nodes in the forced vibration duration. Still taking the Lagrange basis functions as an example, the calculation process can be divided into three cases based on the position of the i-th node:

Case 1, when i∈[1,(l−1)/2],j∈[1,l]

(29) $${Wi}_{j}=\{\begin{array}{l} \frac{\prod_{\begin{array}{l} r=1\\ r\neq i,j \end{array}}^{l}\left(x_{i}-x_{r}\right)}{\prod_{\begin{array}{l} r=1\\ r\neq j \end{array}}^{l}\left(x_{j}-x_{r}\right)},j\neq i\\ \sum_{\begin{array}{l} r=1\\ r\neq i \end{array}}^{l}\frac{1}{\left(x_{i}-x_{r}\right)},j=i \end{array}$$

Case 2, when i∈[(l+1)/2,m+1−(l−1)/2],j∈[i−(l−1)/2,i+(l−1)/2]

(30) $${Wi}_{j}=\{\begin{array}{l} \frac{\prod_{\begin{array}{l} r=i-\left(l-1\right)/2\\ r\neq i,j \end{array}}^{i+\left(l-1\right)/2}\left(x_{i}-x_{r}\right)}{\prod_{\begin{array}{l} r=i-\left(l-1\right)/2\\ r\neq j \end{array}}^{i+\left(l-1\right)/2}\left(x_{j}-x_{r}\right)},j\neq i\\ \sum_{\begin{array}{l} r=i-\left(l-1\right)/2\\ r\neq i \end{array}}^{i+\left(l-1\right)/2}\frac{1}{\left(x_{i}-x_{r}\right)},j=i \end{array}$$

Case 3, when i∈[m+2−(l−1)/2,m+1],j∈[m+2−l,m+1]

(31) $${Wi}_{j}=\{\begin{array}{l} \frac{\prod_{\begin{array}{l} r=m+2-l\\ r\neq i,j \end{array}}^{m+1}\left(x_{i}-x_{r}\right)}{\prod_{\begin{array}{l} r=m+2-l\\ r\neq j \end{array}}^{m+1}\left(x_{j}-x_{r}\right)},j\neq i\\ \sum_{\begin{array}{l} r=m+2-l\\ r\neq i \end{array}}^{m+1}\frac{1}{\left(x_{i}-x_{r}\right)},j=i \end{array}$$

Then a sparse banded coefficient matrix Wn can be generated as shown below.

(32) $$Wn=\left[\begin{array}{ccccccccc} W_{1,1} & W_{1,2} & \cdots & W_{1,l} & & & & & \\ \vdots & \vdots & \vdots & \vdots & & & & & \\ W_{\left(l-1\right)/2,1} & W_{\left(l-1\right)/2,2} & \cdots & W_{\left(l-1\right)/2,l} & & & & & \\ & W_{\left(l+1\right)/2,2} & W_{\left(l+1\right)/2,3} & \cdots & W_{\left(l+1\right)/2,l+1} & & & & \\ & \ddots & \ddots & \ddots & \ddots & \ddots & \ddots & \ddots & \\ & & & & W_{m+1-\left(l-1\right)/2,m+1-l} & \cdots & W_{m+1-\left(l-1\right)/2,m-1} & W_{m+1-\left(l-1\right)/2,m} & \\ & & & & & W_{m+2-\left(l-1\right)/2,m+2-l} & \cdots & W_{m+2-\left(l-1\right)/2,m} & W_{m+2-\left(l-1\right)/2,m+1}\\ & & & & & \vdots & \vdots & \vdots & \vdots \\ & & & & & W_{m+1,m+2-l} & \cdots & W_{m+1,m} & W_{m+1,m+1} \end{array}\right]$$

Replacing the old coefficient matrix W in Section 3.2 with Wn yields a new Floquet transition matrix as follows:

(33) $$\Phi_{2}=P_{2}^{-1}\cdot Q_{2}$$

where, $P_{2}=\Psi -Wn$ , $Q_{2}=Q_{1}$ , and

(34) $$\Psi =\left[\begin{array}{cccc} A+B_{1} & & & \\ & A+B_{2} & & \\ & & \ddots & \\ & & & A+B_{m+1} \end{array}\right]$$

Then, the stability of the milling process can be determined based on the Floquet theory. Obviously, selecting l local nodes significantly reduces the number of interpolation nodes, which implies that the LDQM algorithm can effectively improve the numerical stability of the classical DQM.

It should be noted that parameter l is a variable parameter that can be selected according to actual needs. In addition, although the above LDQM is obtained based on the Lagrange basis functions, other kinds of interpolation basis functions can also be used to construct the corresponding LDQM.

## 4. Numerical Validation and Discussion

The calculation accuracy and efficiency of the proposed method are verified on account of two benchmark milling models in this Section.

Firstly, a single-DOF milling model [21,40] is used to verify the effectiveness and convergence rate of the method, the governing equation of the milling model is represented as follows:

(35) $$\ddot{x}\left(t\right)+2\zeta \omega_{n}\dot{x}\left(t\right)+\omega_{n}^{2}x\left(t\right)=\frac{w\cdot h_{xx}\left(t\right)}{m_{t}}\left(x\left(t\right)-x\left(t-T\right)\right)$$

The values of the parameters in this milling model are obtained through dynamic and cutting experiments. The selected cutting tool is a 12.7 mm diameter (11.8 mm relieved shank diameter) two flute, helical carbide end mill with a 104 mm overhang from the collet holder face (HSK 63A holder/spindle interface). More details of the milling model can be found in Ref. [40]. The values of the dynamics parameters of the milling model are shown in Table 1. When converting Equation (35) to the state-space form represented by Equation (5), the state vector can be represented as $x={\left[\begin{array}{cc} x & \dot{x} \end{array}\right]}^{T}$ , and matrixes A and B are shown as follows:

(36) $$A=\left[\begin{array}{cc} 0 & 1\\ -\omega_{n}^{2} & -2\zeta \omega_{n} \end{array}\right]$$

(37) $$B\left(t\right)=\left[\begin{array}{cc} 0 & 0\\ \frac{w\cdot h_{xx}\left(t\right)}{m_{t}} & 0 \end{array}\right]$$

**Figure 3:**
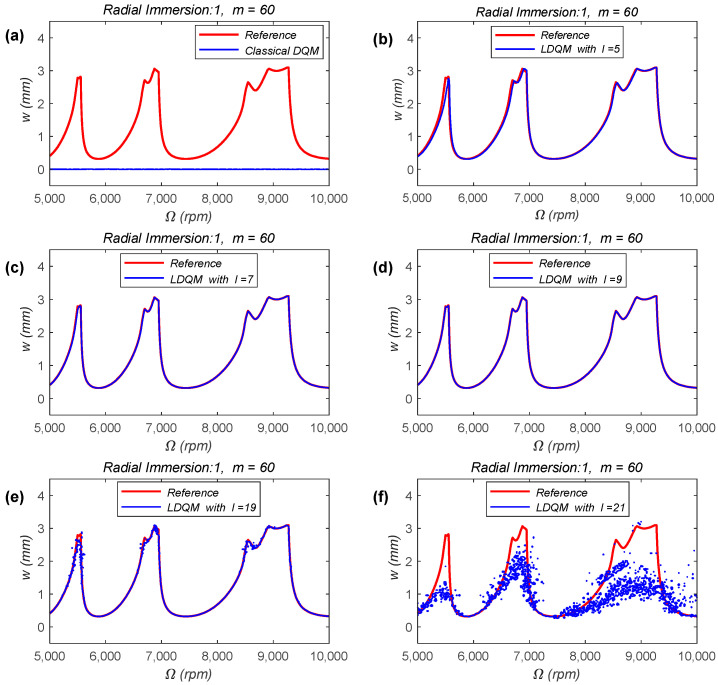
SLD of the single-DOF milling model obtained by classical DQM, and LDQM with different values of parameter l , (**a**) classical DQM; (**b**) LDQM with l=5 ; (**c**) LDQM with l=7 ; (**d**) LDQM with l=9 ; (**e**) LDQM with l=19 ; (**f**) LDQM with l=21 .

The SLD constructed using the LDQM is provided to verify the effectiveness of the algorithm. Here we use the SLD constructed via 1st-SDM as the benchmark, as it has been experimentally validated and widely utilized. The SLD of the single-DOF milling model in this Section is constructed over a 200 × 100-sized grid, and the forced vibration duration in one cutting period is discretized into 60 uniform grids, that is, the parameter m is selected as 60. The machining condition is down-milling, the radial immersion ratio is set as 1 and the simulation parameters are set as follows: the spindle speed ranges from 5000 to 10,000 rpm, and the axial depth of cutting ranges from 0 to 4 mm. The results are shown in Figure 3, in the SLD, the reference stability limits represented by the red line are obtained by the 1st-SDM with m=200 .

Figure 3a–f show the SLD obtained using classical DQM and LDQM with different values of parameter l , respectively. Among them, Figure 3a shows the SLD obtained using classical DQM, while Figure 3b–f show the SLD obtained using LDQM with the local parameter l being selected as 5, 7, 9, 19, and 21. As previously analyzed, classical DQM is prone to generating ill-conditioned coefficient matrices when a large number of nodes are used, which will affect the stability of the calculation results. This phenomenon is intuitively reflected in Figure 3a. Compared with the reference results, the stability limits obtained using the classical DQM, represented by the blue line in Figure 3a, is a completely incorrect result. However, the results from Figure 3b–d indicate that LDQM can effectively compensate for the deficiency of the classical DQM, resulting in completely correct stability limits. Meanwhile, as the local parameter l increases, the calculation accuracy of LDQM also improves. However, it is worth noting that as the local parameter l further increases, the SLD obtained using LDQM exhibits unstable oscillations, as shown in Figure 3e,f. Moreover, the larger the local parameters l is, the more serious the oscillation of the SLD is. This phenomenon once again confirms that when the number of nodes is large, the weighted coefficient matrix will become ill-conditioned. At the same time, this phenomenon also shows that the choice of local parameter l has a significant impact on the calculation results of LDQM, and also provides ideas for the selection of the local parameter.

**Figure 4:**
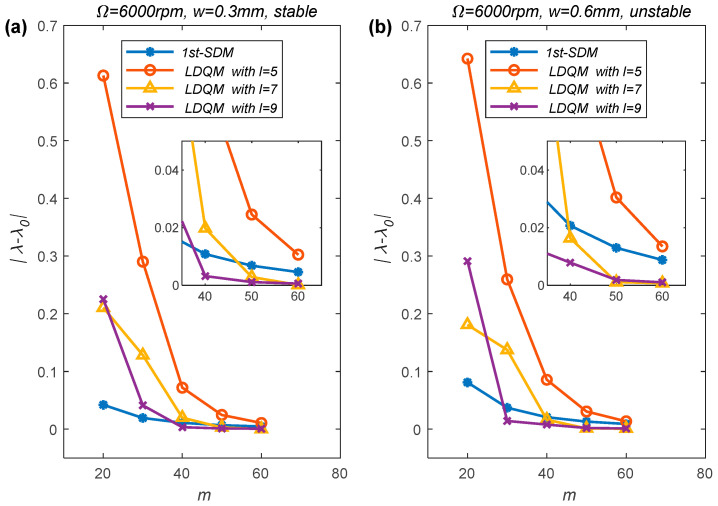
Convergence rate comparisons of the 1st-SDM and LDQM with different values of parameter l , (**a**) stable processing condition, w=0.3 mm ; (**b**) unstable processing condition, w=0.6 mm .

Next, the convergence rate of LDQM is also studied on the basis of the single-DOF milling model and compared with that of the 1st-SDM through numerical simulation. Let $\lambda_{0}$ denote the exact value of the spectral radius of the Floquet transition matrix, λ denote the approximate value of the spectral radius of the Floquet transition matrix obtained using 1st-SDM or LDQM. The difference between λ and $\lambda_{0}$ is represented as a discrete function in terms of the discrete parameter m . The simulation condition is down-milling, the radial immersion ratio is set as 1 and the spindle speed is selected as 6000 rpm, two axial depths ( w=0.3 mm and w=0.6 mm ) of cutting under the stable and unstable processing conditions are selected, and the exact value of $\lambda_{0}$ is determined via 1st-SDM with m=200 . The numerical simulation results are displayed in Figure 4.

From Figure 4, we can discover that the spectral radius λ obtained using the 1st-SDM and LDQM with l=5,7,9 all converging to $\lambda_{0}$ with the increase of the number of the discrete grid m . However, the spectral radius obtained using LDQM approaches the exact value $\lambda_{0}$ significantly faster than that obtained using 1st-SDM. Meanwhile, the calculation accuracy of LDQM increases with the increase of the local parameter l , which is consistent with the conclusion drawn from the SLD in Figure 3. From the locally enlarged images in Figure 4a,b, it can be seen that when m is less than 60, the calculation accuracy of LDQM is lower than the 1st-SDM, while when l is 7 or 9, the calculation accuracy of LDQM is higher than that of the 1st-SDM when m is greater than a certain value. From the trend of the convergence rate curve, it can be seen that as m increases, the calculation accuracy of LDQM under different parameters will ultimately be higher than that of the 1st-SDM.

To further verify the effectiveness of LDQM, a two-DOF milling model [21] is also studied. Similar to the single-DOF milling model, we can define a new state vector $x={\left[\begin{array}{cccc} x & y & \dot{x} & \dot{y} \end{array}\right]}^{T}$ and convert the dynamic equations of the two-DOF milling model into the state-space form shown in Equation (5). Then, we get

(38) $$A=\left[\begin{array}{cccc} 0 & 0 & 1 & 0\\ 0 & 0 & 0 & 1\\ -\omega_{n}^{2} & 0 & -2\zeta \omega_{n} & 0\\ 0 & -\omega_{n}^{2} & 0 & -2\zeta \omega_{n} \end{array}\right]$$

(39) $$B\left(t\right)=\left[\begin{array}{cccc} 0 & 0 & 0 & 0\\ 0 & 0 & 0 & 0\\ \frac{w\cdot h_{xx}\left(t\right)}{m_{t}} & \frac{w\cdot h_{xy}\left(t\right)}{m_{t}} & 0 & 0\\ \frac{w\cdot h_{yx}\left(t\right)}{m_{t}} & \frac{w\cdot h_{yy}\left(t\right)}{m_{t}} & 0 & 0 \end{array}\right]$$

All the parameters in the two-DOF milling model have the same meanings and values as those mentioned in the single-DOF milling model.

**Figure 5:**
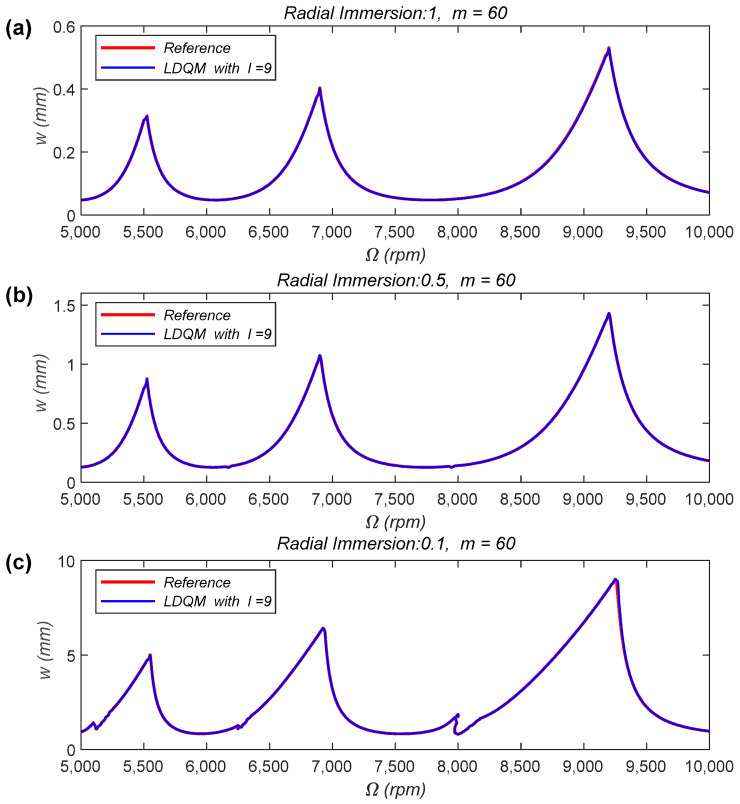
SLD of the two-DOF milling model obtained by LDQM, (**a**) radial immersion 1, m=60 ; (**b**) radial immersion 0.5, m=60 ; (**c**) radial immersion 0.1, m=60 .

The SLD of the two-DOF milling model constructed with the LDQM are provided as well. The SLD are constructed over a 200 × 100-sized grid and the machining condition is set as down-milling, the spindle speed ranges from 5000 to 10,000 rpm and the axial depth of cutting ranges from 0 to 10 mm. The results are plotted in Figure 5, in the SLD, the reference stability limits represented by the red line are obtained using the 1st-SDM with m=200 .

In Figure 5a–c, the radial immersion ratios are 1, 0.5 and 0.1, respectively. The number of the discrete grid m is selected as 60. It can be seen from Figure 5 that under the above three cutting conditions, the stability limits calculated by LDQM with l=9 show perfect agreement with the reference stability limits.

The results of Figure 3 and Figure 5 indicate that the milling stability analysis method proposed in this paper is not limited by the DOF of the milling system or the milling conditions. At the same time, it applies to milling conditions with both large and small radial depths of cutting.

Finally, the computation efficiency of the proposed method is also studied and compared with that of the 1st-SDM with the two-DOF milling model. The simulative calculations are performed in Matlab^®^ on a desktop computer (Intel^®^ Core^TM^ i7-10700F, 2.90 GHz, 16.00 GB RAM). Here, three different radial immersion ratios (1, 0.5 and 0.1) are selected and the number of the discrete grid m is set as 60. The calculation time of the 1st-SDM and LDQM with l=5,7,9 are listed in Table 2.

As outlined in Table 2, the time consumption of the LDQM with three different parameters are close to each other. At the same time, it can be seen from the MATLAB profiler that the time of the LDQM is mainly consumed in the calculation of the spectral radius. Compared with the 1st-SDM, the LDQM can save up to 80% of the calculation time. This result shows that besides the calculation accuracy, the proposed method also has high computation efficiency.

## 5. Conclusions

This paper is dedicated to the analysis of chatter stability in milling processes. A novel method based on LDQM is developed to study the chatter stability of milling processes. The proposed method represents the derivative of the state vector in the milling dynamics equation as the weighted sum of state vector values at local nodes and simplifies the differential equations in the state space into algebraic equations, thereby easily constructing the Floquet transition matrix during one tooth passing period. Finally, the stability of the milling process is obtained based on the Floquet theory. Simulation analysis of the benchmark milling models confirms that the method proposed in this paper is suitable for various milling conditions and has good accuracy and computational efficiency. The advantages of the method proposed in this work are as follows:

1. Using the LDQM to process the milling dynamics equation can generate a sparse weighted coefficient matrix, which effectively improves the stability of numerical calculations.
2. The local parameter l in the proposed method can be flexibly adjusted as needed to achieve both the computational accuracy and efficiency.
3. The method presented in this work is not limited by the DOF of the milling system or the milling conditions and is suitable for both large and small radial depths of cutting.

## Figures and Tables

**Table 1:** Values of the dynamics parameters of the milling model.

*m_t_* (kg)	*ζ*	*ω_n_* (rad/s)	*K*_tc_ (N/m^2^)	*K*_rc_ (N/m^2^)	*N*
0.03393	0.011	5793	6 × 10^8^	2 × 10^8^	2

**Table 2:** Calculation time required for the 1st-SDM and LDQM with different values of parameter l (unit: s).

m=60	Radial Immersion: 1	Radial Immersion: 0.5	Radial Immersion: 0.1
1st-SDM	255.70	251.25	248.25
LDQM (l=5)	41.89	38.86	37.45
LDQM (l=7)	41.97	37.29	36.65
LDQM (l=9)	42.97	37.27	36.03

## Data Availability

Data are contained within the article.
